# Evidence for bacteriophage T7 tail extension during DNA injection

**DOI:** 10.1186/1756-0500-1-36

**Published:** 2008-06-26

**Authors:** Philip Serwer, Elena T Wright, Kevin W Hakala, Susan T Weintraub

**Affiliations:** 1 Department of Biochemistry, The University of Texas Health Science Center, San Antonio, Texas 78229-3900, USA

## Abstract

**Background:**

Electron micrographs of bacteriophage T7 reveal a tail shorter than needed to reach host cytoplasm during infection-initiating injection of a T7 DNA molecule through the tail and cell envelope. However, recent data indicate that internal T7 proteins are injected before the DNA molecule is injected. Thus, bacteriophage/host adsorption potentially causes internal proteins to become external and lengthen the tail for DNA injection. But, the proposed adsorption-induced tail lengthening has never been visualized.

**Findings:**

In the present study, electron microscopy of particles in T7 lysates reveals a needle-like capsid extension that attaches partially emptied bacteriophage T7 capsids to non-capsid vesicles and sometimes enters an attached vesicle. This extension is 40–55 nm long, 1.7–2.4× longer than the T7 tail and likely to be the proposed lengthened tail. The extension is 8–11 nm in diameter, thinner than most of the tail, with an axial hole 3–4 nm in diameter. Though the bound vesicles are not identified by microscopy, these vesicles resemble the major vesicles in T7 lysates, found to be *E. coli *outer membrane vesicles by non-denaturing agarose gel electrophoresis, followed by mass spectrometry.

**Conclusion:**

The observed lengthened tail is long enough to reach host cytoplasm during DNA injection. Its channel is wide enough to be a conduit for DNA injection and narrow enough to clamp DNA during a previously observed stalling/re-starting of injection. However, its outer diameter is too large to explain formation by passing of an intact assembly through any known capsid hole unless the hole is widened.

## Findings

A double-stranded DNA bacteriophage starts an infection by injecting its DNA genome into a host cell; progeny later assemble by packaging a genome. The protein movements that cause DNA injection are usually assumed to be restricted primarily to an external tail; the protein movements of DNA packaging are usually assumed to be restricted primarily to a multimeric DNA packaging ATPase [reviewed in references [1-3]]. However, as previously discussed [4], the protruding portion of the tail of bacteriophage T7 (23 nm long [5]) appears not long enough to reach from the outer shell of the bacteriophage, through the outer host membrane, the periplasmic space/cell wall and then the inner membrane, total distance about 24 nm [6,7]. Thus, DNA injection appears to require lengthening of the T7 tail.

**Figure 1:**
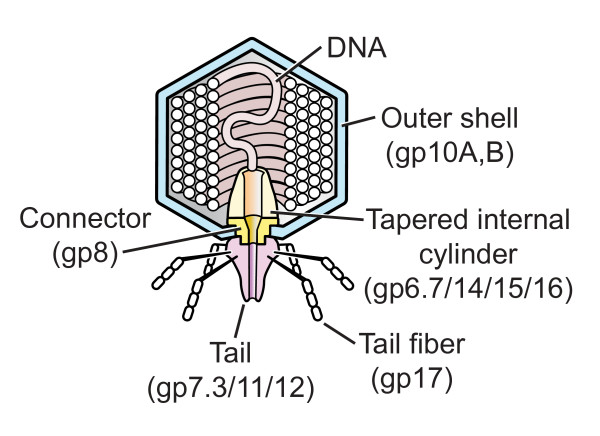
**The structure of bacteriophage T7 **[9,11,24]. The internal tapered cylinder and tail are shown attached to an icosahedral outer shell by a connector.

Tail lengthening is supported by the following observations: (a) After bacteriophage/host binding, five bacteriophage proteins are detected within the host cell before the DNA molecule is injected: gp6.7, gp7.3, gp14, gp15 and gp16 [8,9]; T7 proteins are named by gp, followed by the gene number, as previously reviewed [10]. Of these "injected" proteins, all but gp7.3 [9] are part of an internal, tapered protein cylinder [11] that has an axial hole, is co-axial with the T7 tail and is surrounded by the packaged genome before injection (Figure 1). Injection of internal proteins is not unique to T7-class bacteriophages; injection of internal bacteriophage T4 proteins has also been demonstrated [12]. (b) After the first 850–1000 DNA base pairs (with a length of 289–340 nm), T7 DNA injection stops and does not re-start until transcription initiated by an injected promoter re-starts injection. The DNA initially injected is protected from host nucleases [13,14]. The mechanism of this protection is not known [4,13,14].

The injected internal proteins potentially lengthen the T7 tail for DNA injection and also possibly DNA protection after injection [8,15]. However, evidence for such tail lengthening was not reported in the studies of references 4, 8, 9, 13 and 14. Intriguingly, a capsid has been observed with needle-like extension long enough to be a lengthened T7 tail [15]. However, the extension was not bound to outer membrane, as it would be if injecting DNA. In the present study, a similar extension is observed by electron microscopy to attach and sometimes penetrate vesicles likely to be from the outer host membrane.

**Figure 2:**
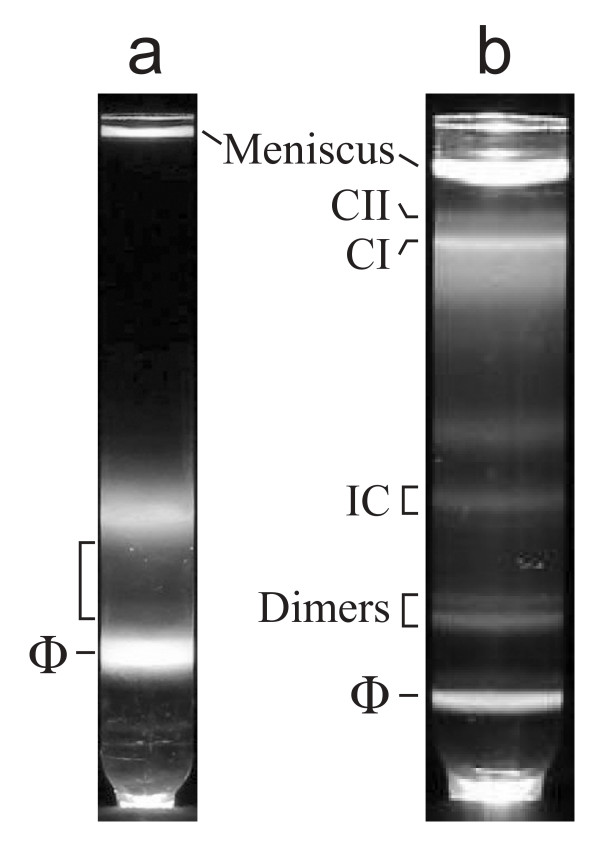
**Fractionation of a lysate of bacteriophage T7-infected *E. coli***. Particles in a 6-liter lysate of bacteriophage T7-infected *E. coli *BL21 were precipitated with polyethylene glycol, clarified by low speed centrifugation and DNase-digested. (a) The particles were then layered on a cesium chloride step gradient and centrifuged [11]. Light scattering from particles in the gradient was photographed; a bacteriophage particle band is indicated by ϕ. (b) Particles in the bracketed region of the step gradient were brought to a density of 1.367 g/ml with cesium chloride and centrifuged to equilibrium in a Beckman SW55 rotor at 42,000 rpm for 22.0 hr. at 4°C. Light scattering from the gradient was photographed. Fractions were dialyzed against 0.2 M NaCl, 0.01 M Tris-Cl, pH 7.4, 0.001 M MgCl_2_. The dialyzed IC fraction was the sample used for electron microscopy in Figures 3 and 4, below.

The sample for electron microscopy was obtained with a procedure of reference 11, briefly: Particles in a 6-liter lysate of T7-infected *E. coli *were concentrated by polyethylene glycol precipitation. The precipitate was resuspended and centrifuged through a cesium chloride step gradient (profile of light scattering: Figure 2a). The fraction of the step gradient indicated by a bracket in Figure 2a was subjected to buoyant density centrifugation in a cesium chloride density gradient (profile of light scattering: Figure 2b). The density gradient of Figure 2b had the following features: The lower of two bands near the top of the gradient was produced by the T7 procapsid (also called capsid I; labeled CI in Figure 2b; density = 1.283 g/ml). Capsid I appears before DNA packaging occurs and is converted to a more bacteriophage-like capsid (capsid II) during DNA packaging [1,3]. The second band near the top of the gradient was produced by capsid II (labeled CII in Figure 2b; density = 1.272 g/ml) (capsid identification not shown). Lower bands were produced by dimeric capsid/bacteriophage particle aggregates (dimer; density = 1.398 and 1.411 g/ml) in Figure 2b; the lowest band was produced by bacteriophage particles (ϕ in Figure 2b).

Electron microscopy revealed that the region between CII and dimers in Figure 2b had vesicle-like particles, possibly host outer membrane vesicles [16,17]. Thus, gradient fractions were screened for bacteriophage capsids bound to vesicle-like particles in a complex that had the following characteristics expected of a DNA injection complex (IC): (a) either a bacteriophage tail or a lengthened tail connected bacteriophage to vesicle-like particle (to be called a vesicle) and (b) DNA was at least partially emptied from the capsid. Apparent IC's were found concentrated in the region of the gradient indicated with the bracket labeled IC and coincident with a band of light scattering in Figure 2b (1.359 g/ml).

**Figure 3:**
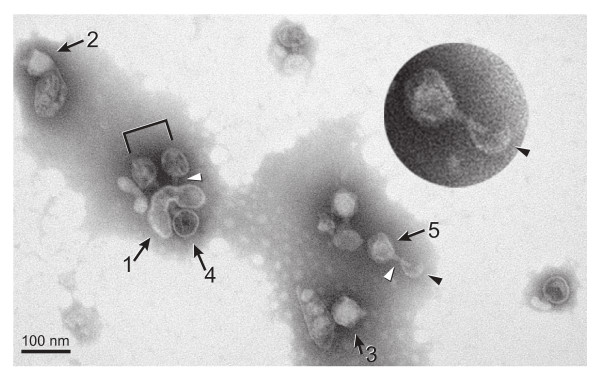
**Electron microscopy: a single field of particles.** The IC fraction from Figure 2b was prepared for electron microscopy by negative staining with 1% sodium phosphotungstate, pH 7.6 [11]. A single field of particles is shown that has several potential IC's. The length of the bar is 100 nm. The variability of the thickness of the negative stain is larger than sometimes observed, because of the extent of both the size variability and the diluteness of the particles of the specimen.

These apparent IC's each consisted of (a) a vesicle of variable size and shape and (b) one or more icosahedral T7 capsids. Figure 3 has an electron microscope field with several apparent IC's. Arrow 1 in Figure 3 indicates a non-spherical vesicle that, at the top, has bound two T7 capsids (indicated by a bracket), both apparently vesicle-attached via a needle-like capsid extension (white arrowheads) that is slightly (10–15%) longer than the 23 nm T7 tail. Both these capsids also appear to have internal material. The amount of the internal material is less than in mature bacteriophage particles, based on comparison to the bacteriophage particles indicated by arrows 2 and 3. However, the bracket-marked capsids were not empty, based on comparison to the apparently empty capsid indicated by arrow 4. Thus, the conclusion is drawn that the bracket-marked capsids have DNA, but in amount less than the amount in a bacteriophage particle, as expected for an IC.

The bacteriophage particles indicated by arrows 2 and 3 in Figure 3 have tails (note the tapering) and are attached via their DNA-containing outer shells (not the tails) to an object previously characterized [5,18] as a product (called polycapsid) of mis-assembly of the major protein (gp10) of the capsid's outer shell. The polycapsids have vesicles within them, a point previously noted [5,18]. The polycapsid-adsorbed bacteriophages have not injected DNA and this adsorption is assumed not to have significance for biological injection.

For the two bracket-marked capsids in Figure 3, the needle-like extension appears less tapered than the tail, an observation also made for all 27 other vesicle attached extensions observed. The total extension length cannot be determined for the bracket-marked capsids because of partial obscuring of part of the extension, especially by material inside of the vesicle. Although partially obscured by this material, the extension of the left bracket-marked capsid in Figure 3 does appear to extend in a straight line through the proximal surface of the vesicle, completely to the distal vesicle surface, producing a total length of 40–50 nm.

**Figure 4:**
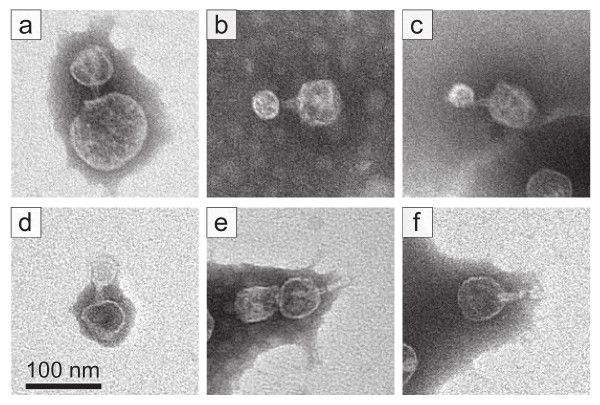
**Electron microscopy: selected particles.** (a) – (d) Bacteriophage capsids bound to a vesicle via a needle-like extension that is thinner and, with the exception of (a), visibly longer than the T7 tail. (e) A longer-than-tail, needle-like extension-containing capsid not attached to a vesicle. (f) A capsid particle with an apparently broken needle-like extension. The length of the bar is 100 nm.

The needle-like extension associated with the capsid indicated by arrow 5 of Figure 3 (magnified in the inset) is seen more clearly to penetrate the attached vesicle and has a length of 41 nm, but also appears to have an additional ~14 nm broken from the extension at the distal inner surface of the vesicle (black arrowhead in Figure 3 and inset). The high visibility within the vesicle is apparently caused by vesicle breakage/leakage that is indicated by the discontinuity of the vesicle's boundary. The capsid of this complex is partially full of DNA. The outer diameter of the needle is 8–11 nm, significantly smaller than the diameter of the wider end of the tail. A central channel, 3–4 nm in diameter, is sometimes visible, as illustrated by the more magnified complexes in Figure 4a–d. Thirty-five needle-like extensions, all with diameter in this range, were observed. Some were on capsids that were not attached to vesicles. Some had lengths of 50–60 nm (Figure 4e). Most had extensions that were shorter, possibly broken (Figure 4f).

When analyzed by non-denaturing agarose gel electrophoresis, the vesicle-containing fractions of the gradient in Figure 2b all produced a single, broad "vesicle" band that was further from the origin than capsids (not shown). To characterize the vesicles, vesicle proteins were analyzed after vesicle purification by both the centrifugation of Figure 2a, b and then preparative non-denaturing two-dimensional agarose gel electrophoresis (2d-AGE). The sample for 2d-AGE was a solution of Alexa 488-stained particles with a density of 1.265 g/ml (density of capsid II). The procedure of 2d-AGE uses a dilute (0.3%) gel for the first dimension and a more concentrated (1.8%) gel for the second; details are described and reviewed in reference 19.

**Figure 5:**
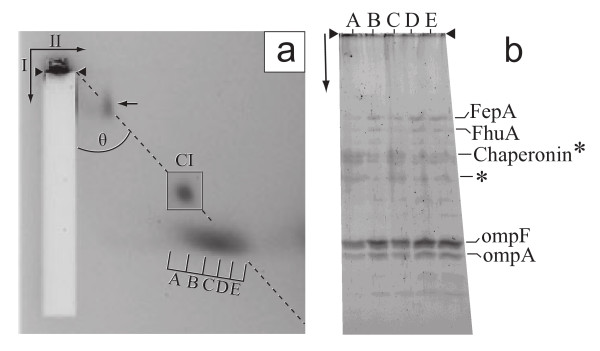
**Analysis of host vesicles**. (a) Host vesicles were prepared as in Figure 2, but using a lysate of *E. coli *BL21 infected with T7 amber mutant in genes 5 (DNA polymerase) and 19 (DNA packaging ATPase). Proteins in a fraction with density of 1.265 g/ml were then stained with 80 μg/ml Alexa 488 at 21°C for 2 hr. in 0.09 M Tris-Acetate, pH 8.4, 0.001 M MgCl_2 _(electrophoresis buffer). Alexa-stained particles were subjected to 2d-AGE [19] in electrophoresis buffer at room temperature (25 ± 3°C) through one of four 0.3% first dimension gels that were embedded in a 1.8% second dimension gel [19]. First dimension: 2.0 V/cm, 3.8 hr; second dimension: 1.8 V/cm, 8.5 hr. Alexa 488 fluorescence was observed under ultraviolet illumination, photographed and used for slicing the agarose gel (slices A-E). Capsid I (radius = 26.1 nm) was fractionated in another first dimension gel embedded in the same second dimension gel. The band of capsid I (CI), positioned accurately, is overlaid in a box. Arrowheads indicate origin; arrows outside of the gel indicate directions of first (I) and second (II) dimension electrophoresis. The dashed line is drawn from the origin through the CI band; particles on this line have *R*_E _of 26.1 nm. (b) The slices described above were subjected to SDSPAGE in a 10% gel and stained with SYPRO Ruby protein gel stain (Bio-Rad). Proteins from selected bands were digested with trypsin and analyzed by capillary HPLC-tandem mass spectrometry, as previously described [25]. Protein identity is indicated at the right. The proteins marked with an asterisk were present in the background, as well as the stained agarose gel regions, for reasons not known. The presence in the background of a chaperonin is, however, not surprising because of the known non-specific and reversible protein-chaperonin binding.

The 2d-AGE fractionates primarily by average electrical surface charge density (σ) in the first dimension and by both σ and effective radius (*R*_E_) in the second dimension. The value of *R*_E _is a decreasing function of the angle (θ) subtended by the direction of the first field and a line drawn from the origin to the position of a particle in the gel. The line for a *R*_E _of 26.1 nm is drawn in Figure 5a. For this analysis, the vesicles were produced by use of a T7 mutant that assembles capsid I, but does not package DNA and does not convert capsid I to capsid II. Thus, complications from the presence of bacteriophage particles were minimized.

The fluorescence profile of Alexa 488-stained particles after 2d-AGE had primarily two features. (a) A band of a capsid not further investigated had a small percentage of the fluorescence (arrow inside Figure 5a). (b) A streak of vesicles had most fluorescence (bracket with capital letters in Figure 5a). The *R*_E _standard is capsid I (boxed band in Figure 5a). The capsid I had been run in parallel in a gel embedded in the same agarose frame (different quadrant) in which the gel of Figure 5a was embedded [19]; the image of the capsid I band was then overlaid on the image of Figure 5a. A streak of the type observed above for vesicles is expected for particles that are uniform in σ and variable in *R*_E _[19]. The range of *R*_E _values for streak-producing particles was determined by quantification of θ values at the two ends of the streak and was found to be 20–50 nm, within the range of values previously found for *E. coli *outer membrane vesicles (10–100 nm [17]).

For further analysis, the gel at the position of the streak of Figure 5a was cut into five slices, indicated by letters in Figure 5a. The slices were boiled and the proteins were analyzed by sodium dodecylsulfate polyacrylamide gel electrophoresis (SDSPAGE). After staining with SYPRO Ruby protein gel stain (Figure 5b), the proteins of the indicated bands were excised, digested *in situ *with trypsin and identified by HPLC-electrospray-tandem mass spectrometry. The two major proteins identified for slices A-E were ompA [20] and ompF [21], as indicated in Figure 5b; these proteins were not detected in several non-staining areas of the agarose gel of Figure 5a that were analyzed (not shown).

The next most abundant protein was the 60 Kd chaperonin (indicated by chaperonin in Figure 5b). However, this protein was found in several locations in the agarose gel, even where no band or streak was detected. The 60 Kd chaperonin is, therefore, not significantly associated with the vesicles. Additional, vesicle-associated proteins were ferrichrome iron receptor protein, product of the FhuA gene (FhuA in Figure 5b) [22] and ferrienterobactin outer-membrane receptor precursor, product of the FepA gene (FepA in Figure 5b) [23]. OmpA, ompF, FhuA and FepA proteins are all *E. coli *outer membrane proteins. Thus, we conclude that the vesicles are formed from the *E. coli *outer membrane.

The length of the observed needle-like extension is great enough to more than traverse the host's outer membrane, periplasmic space/cell wall and inner membrane. Thus, we hypothesize that this extension lengthens the tail for DNA injection and that the extension contains at least some of the proteins that others [8,9] have found to be injected. The small size of its channel supports the hypothesis [14] that a constriction causes the pre-transcription delay of injection. The extension is too short to directly explain protection of the initially injected 850–1,000 base pairs.

If gp6.7, gp14, gp15 and gp16 are part of the lengthened tail, the question remains: Did these proteins cross the capsid's outer shell while assembled as in Figure 1? If so, were the assembled proteins unfolded, as previously proposed [8]? Or, did these proteins cross as single protein molecules and subsequently assemble to form the needle? If the proteins cross while both assembled and folded, the outer shell/tail connector (a ring of gp8; Figure 1) would have to widen its central hole that varies from 3.5–4.5 nm in diameter [24]. In either case, formation of the tail extension would require protein motion not previously observed.

## Competing interests

The authors declare that they have no competing interests.

## Authors' contributions

PS conceived the study, designed the preparative protocols, performed the electron microscopy and wrote the manuscript. EW performed the preparative work. KH performed the mass spectrometry. STW directed the mass spectrometry laboratory and assisted in writing the manuscript. All authors read and approved the final manuscript.
